# Epigenetic alterations in triple-negative breast cancer and their clinical implications for diagnosis and therapy

**DOI:** 10.7150/jca.119442

**Published:** 2025-09-22

**Authors:** Eun-Jin Go, Ji Hoon Oh

**Affiliations:** Department of Biological Sciences, Keimyung University College of Natural Sciences, Daegu 42601, Republic of Korea.

**Keywords:** breast cancer, clinical applications, epigenetics, poor survival, triple-negative

## Abstract

Breast cancer, including triple-negative breast cancer (TNBC), is the most commonly diagnosed cancer in women, with subtypes differing in treatment options and prognoses. In particular, TNBC, characterized by the absence of estrogen receptor (ER), progesterone receptor (PR), and human epidermal growth factor receptor 2 (HER2) expression, is the most aggressive subtype, with limited treatment options, high metastatic rates, and poor survival outcomes. In recent years, epigenetic studies have emerged as a promising tool for analyzing gene expression and alterations in TNBC, providing potential insights into the development of novel therapeutic strategies. Epigenetic mechanisms, such as DNA methylation, histone modifications, and non-coding RNA (ncRNA)-mediated gene silencing, play a crucial role in the development and progression of TNBC. Research into these mechanisms holds significant promise for the development of personalized therapeutic approaches, potentially improving outcomes for TNBC patients. This review provides a comprehensive overview of recent advances in research on epigenetic alterations in TNBC, with an emphasis on potential clinical applications aimed at improving survival and quality of life in TNBC patients.

## 1. Introduction

**Figure 1:**
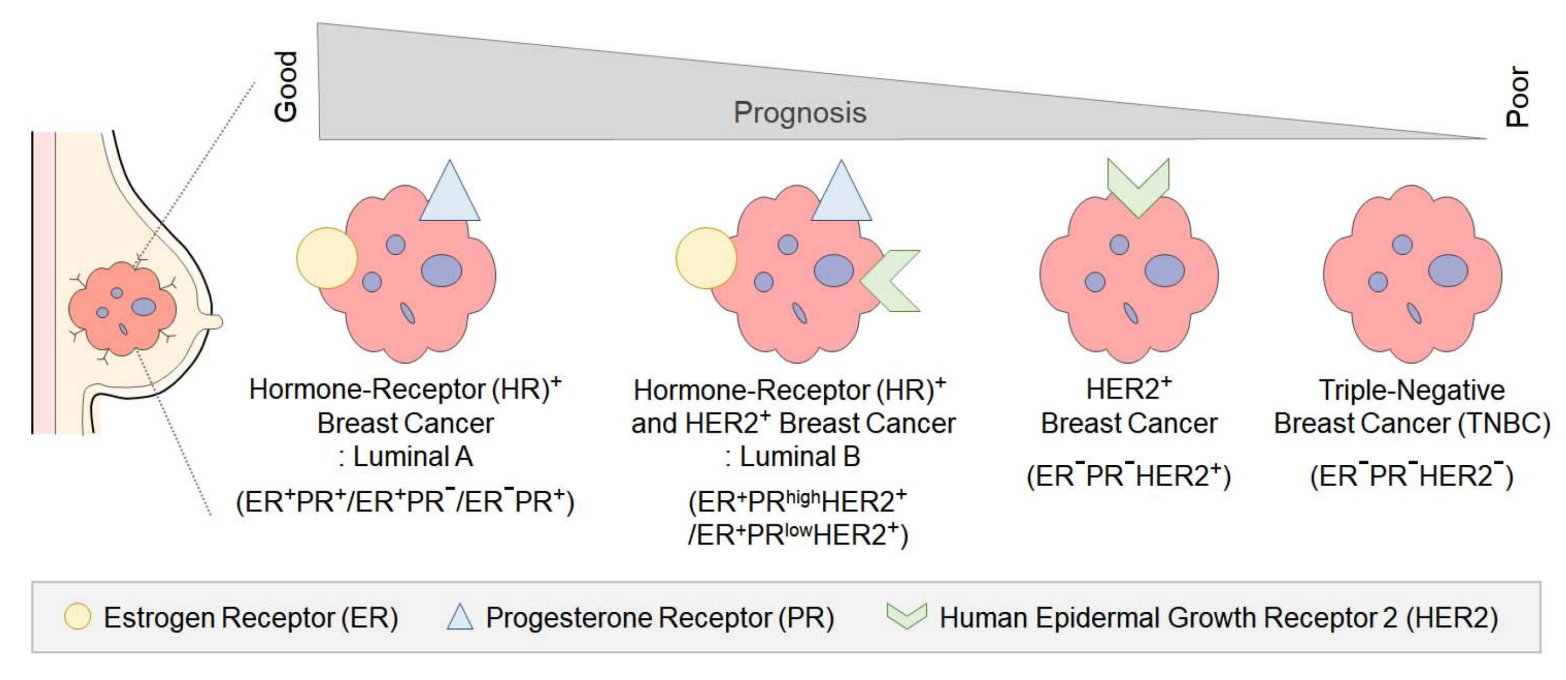
Classification of breast cancer subtypes and prognosis Breast cancer subtypes are classified by the expression of estrogen receptor (ER), progesterone receptor (PR), and human epidermal growth factor receptor 2 (HER2). ER+/PR+/HER2+ tumors show a worse prognosis due to HER2 amplification, which drives aggressive tumor behavior. ER-/PR-/HER2+ tumors exhibit high proliferation rates and aggressive clinical courses due to the lack of hormone receptor expression. Triple-negative tumors (ER-/PR-/HER2-) lack all three receptors, leading to limited treatment options and the poorest prognosis.

Breast cancer is the most frequently diagnosed cancer in women, with a global incidence of over 2.26 million cases in 2020. The classification of breast cancer is based on the expression of specific receptors, namely estrogen receptor (ER), progesterone receptor (PR), and human epidermal growth factor receptor type 2 (HER2) 1 (Figure 1). Each subtype of breast cancer exhibits distinct clinical characteristics, treatment responses, and prognoses 2.

Hormone receptor (HR)-positive breast cancer is the most common subtype, accounting for 60-70% of all breast cancers 3. It is characterized by the expression of ER and PR receptors and generally exhibits a more favorable prognosis compared to other subtypes 3. The primary treatment for HR-positive breast cancer involves various adjuvant antihormonal therapies that block the action of the female hormone estrogen, with additional chemotherapy applied as needed 4.

HER2-positive breast cancer represents approximately 15-20% of all breast cancer patients 5. In normal cells, HER2 is a protein that plays a regulatory role in cell growth, division, and survival 6. However, the overexpression of HER2 in HER2-positive breast cancer accelerates tumor growth and increase the likelihood of metastasis 6. The Food and Drug Administration (FDA) approval of trastuzumab in 1998 represented a pivotal advancement in HER2-positive breast cancer treatment 7, 8, resulting in expanded therapeutic options and improved prognosis 9-11.

Triple-negative breast cancer (TNBC) is a type of breast cancer in which the ER, PR, and HER2 proteins are not expressed 12. TNBC occurs more frequently in younger women and is considered the most aggressive subtype, accounting for approximately 10-15% of all breast cancers 13. Combination chemotherapy regimens, primarily based on anthracyclines and taxanes, are the standard therapeutic approach for TNBC 14. However, the absence of targetable receptors for TNBC significantly constrains the availability of efficacious treatment options 14. Consequently, the prognosis is poor, with a high risk of recurrence and metastasis to the brain and lungs, leading to a five-year survival rate of approximately 10% 1, 14. Despite advancements in treatment approaches over the years, there remains an urgent need for more effective therapies that enable precise diagnosis and extend survival in patients with TNBC.

Epigenetic approaches represent a promising area in cancer research. These approaches systematically analyze the genetic mutation processes in TNBC, focusing on the development of personalized therapies to improve early diagnosis and prognosis prediction 15. Epigenetic research on TNBC targets alterations such as DNA methylation, histone modifications, and non-coding RNA (ncRNA)-mediated gene silencing, enabling precise regulation of gene expression and addressing challenges in areas that are difficult to target with conventional therapies 15.

Therefore, this study provides a comprehensive summary of recent research trends on epigenetic alterations associated with TNBC and offers an in-depth discussion of potential research directions and clinical applications aimed at improving survival and quality of life of TNBC patients.

## 2. Epigenetics

**Figure 2:**
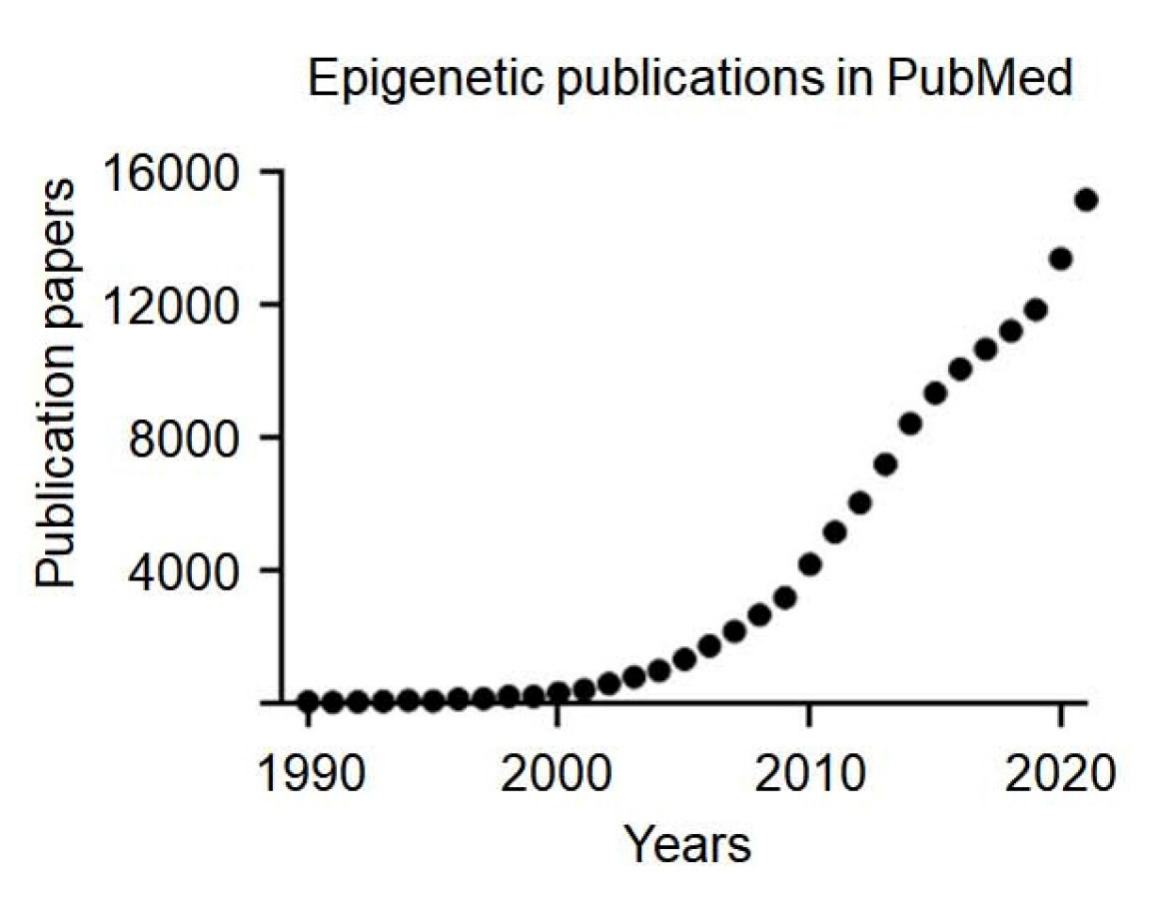
Epigenetic research and lifestyle-induced variation. Epigenetic research growth and lifestyle-induced epigenetic variation. Growth of epigenetics-related publications indexed in PubMed from 1990 to 2020, showing a rapid increase starting in the early 2000s.

Epigenetics is the study of heritable and stable changes in gene expression, occurring through chemical modifications of DNA bases and alterations in chromatin structure, without directly altering the DNA sequence 16. Modifications to specific DNA regions caused by environmental factors are referred to as epigenetic marks, and phenomena that influence gene expression and phenotypes are termed epimutations 17. Since the 2000s, there has been a significant increase in publications on epigenetics (Figure 2A), but limited exploration of its fundamental mechanisms has constrained a comprehensive understanding. Although genetic information is identical within a single organism, the functions and characteristics of tissues or organs are distinctly regulated and specified 18. The differentiation of somatic cells with identical genetic information is regulated by epigenetic mechanisms, leading to differentiation into skin cells, muscle cells, nerve cells, and other specialized cell types 19. Additionally, exposure to various environmental factors, such as age, stress, illness, diet, and smoking, can affect the epigenome 20-22. For example, disease discordance in genetically identical monozygotic twins can be influenced by factors such as environmental exposures, suggesting the involvement of epigenetic factors 23, 24. Epigenetics has emerged as a crucial field in disease and therapeutic development, as its relationship with disease occurrence becomes increasingly evident 22, 25. Particularly in diseases like cancer, research is being conducted to detect abnormal epigenetic changes and to develop therapeutic approaches focused on the epigenome 22. This is expected to contribute not only to cancer treatment, diagnosis, and prevention but also to the development of personalized therapies, playing a crucial role in the future advancement of medicine.

## 3. Therapeutic Potential of Epigenetics

In clinical research, epigenetics is a highly promising field due to its potential to regulate gene expression 19. This facilitates the modulation of disease development and progression without requiring specific genetic modifications 22. Therefore, epigenetic therapies can facilitate personalized treatments based on an individual's genes and epigenetic profile 25. This approach enables the development of more precise and effective treatments while minimizing unnecessary side effects 25. However, safety and ethical considerations must be thoroughly addressed when applying these approaches to humans 25.

Pharmacological agents, such as DNA methyltransferase inhibitors and histone-modifying enzymes, have been extensively investigated, with some demonstrating promising results in clinical trials 19, 26, 27. These agents have shown potential efficacy in addressing complex diseases, including cancer, autoimmune disorders, and neurological diseases 28. The advancement of epigenetic therapies is expected to play a central role in personalized medicine. Advancing the understanding of epigenetic mechanisms and developing novel therapies grounded in these insights are essential to overcoming the limitations of current treatments and enhancing patients' quality of life.

## 4. DNA Methylation in TNBC

**Figure 3:**
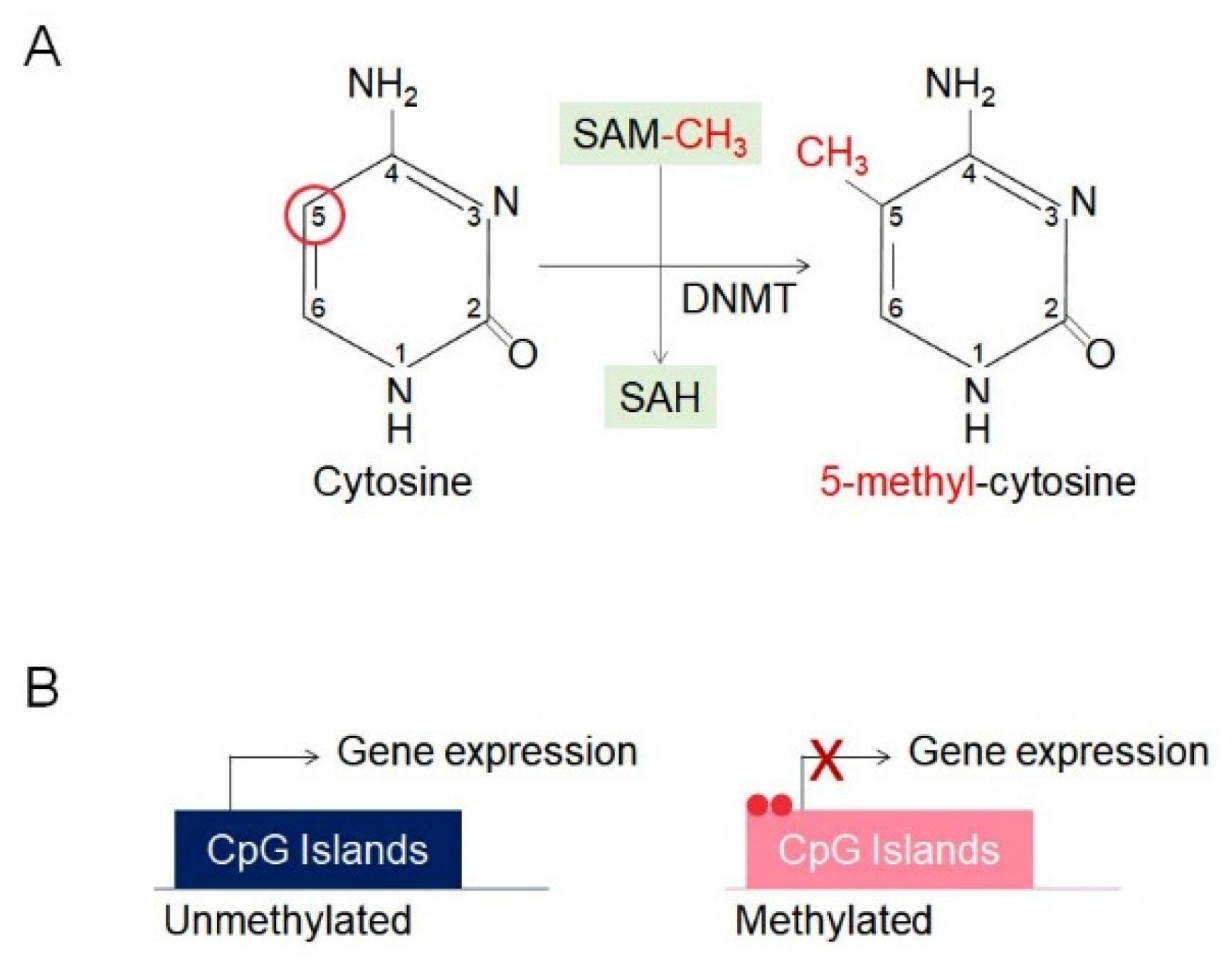
DNA methylation and CpG island regulation. (A) DNA methylation involves the transfer of a methyl group (-CH₃) from S-adenosylmethionine (SAM) to the 5th carbon of cytosine within CpG dinucleotides, catalyzed by DNA methyltransferases (DNMTs), producing 5-methylcytosine (5-mC) and S-adenosylhomocysteine (SAH). (B) CpG island methylation regulates gene expression by silencing transcription. Unmethylated CpG islands allow active transcription, while methylated CpG islands block transcription by preventing access of transcriptional machinery.

DNA methylation, one of the most extensively studied mechanisms in epigenetics, plays a critical role in regulating gene expression by significantly influencing gene function without altering the genetic information 22. Moreover, it is essential for normal development and cellular functions and is strongly associated with the occurrence and progression of various diseases, particularly cancer 22. This process is catalyzed by DNA methyltransferases (DNMTs) and involves the addition of a methyl group (-CH3) to the fifth carbon of the cytosine base, resulting in the formation of 5-methylcytosine (5-mC) 22, 28 (Figure 3A). Predominantly occurring in CpG islands, cytosine-guanine-rich regions, methylation at gene promoters serves to repress gene expression 29, 30 (Figure 3B). Methylated DNA is recognized by methyl-CpG-binding proteins (MBPs) 31, 32. This represses gene expression by preventing transcription factors from binding to DNA or recruiting repressor complexes 33. DNA methylation plays a critical role in tissue-specific gene expression, X-chromosome inactivation, and the precise regulation of gene expression 33. DNA is generally unmethylated at promoter regions, while cytosines are predominantly methylated in normal cells 34. However, certain epigenetic alterations result in promoter hypermethylation, defined by excessive methylation at promoter regions, and global hypomethylation, defined by a reduction in cytosine methylation 34.

Epigenetic alterations in TNBC exhibit distinct methylation patterns when compared with other types of breast cancer, which can suppress the expression of tumor suppressor genes and promote cancer initiation and progression 35-38. In TNBC, DNA methylation-associated genes include pro-apoptotic genes such as the HOX gene family (HOXA, HOXB, HOXC, and HOXD) and TMS1, cell cycle inhibitor genes such as p16 and RASSF1A, and DNA repair genes such as the BRCA family 39. The human HOX gene family comprises 39 genes organized into four clusters 40. Among these, a comparison of HOX gene expression levels in benign and malignant breast cancer tissues revealed that 14 HOX genes (HOXA6, HOXA13, HOXB2, HOXB4, HOXB5, HOXB6, HOXB7, HOXB8, HOXB9, HOXC5, HOXC9, HOXC13, HOXD1, and HOXD8) are overexpressed and are associated with poor prognosis 40. This overexpression of HOX genes in TNBC is often attributed to promoter hypomethylation within CpG islands of HOX gene clusters, particularly in the 5′ regulatory regions of HOXA10 and HOXB13, located on chromosomes 7p15 and 17q21, respectively. Hypomethylation at these sites removes transcriptional repression, leading to aberrant activation of developmental gene programs and epithelial-mesenchymal transition (EMT), which enhances tumor invasiveness and stemness properties 40, 41. A study conducted a comprehensive methylome analysis of TNBC and identified three distinct methylation clusters 42. TNBC patients with low methylation profiles exhibited higher survival rates than those with high methylation profiles 42. Specifically, promoter hypomethylation of HOXA10 and HOXB13 correlates with increased expression of downstream effectors, such as TWIST1 and SNAI2, which are key regulators of EMT and metastasis in TNBC cells. This epigenetic reprogramming distinguishes TNBC from luminal subtypes, where HOX gene methylation is largely preserved 40, 41. However, despite low methylation profiles in some TNBC cases, DNA hypermethylation of BRCA (breast cancer gene) may influence patient prognosis and therapeutic response 42. BRCA genes, including BRCA1 and BRCA2, are associated with a lifetime risk of 60-80% for developing breast cancer in women with mutations in BRCA1 or BRCA2 43, 44. TNBC with BRCA1/2 mutations is referred to as a BRCAness tumor 45. BRCA1 methylation reduces BRCA1 mRNA expression, leading to impaired DNA repair and increased genomic instability 44, 46. BRCA1 methylation is a critical in TNBC occurrence and progression 46. Analysis of breast and leukocyte DNA in over 400 TNBC patients showed that 20% of cancers originated from normal cells with BRCA1 epigenetic alterations 47. When analyzing blood samples from newborns, female infants were found to exhibit epigenetic alterations in BRCA1 associated with TNBC at twice the rate of males 47. These alterations were found to be independent of parental BRCA1 epigenetic modifications 47. This has been reported to involve the overexpression of DNA methyltransferases, including DNMT1, DNMT3A, and DNMT3B 48. Overexpressed DNA methyltransferases exhibit enhanced tumor-promoting properties and are associated with poor prognosis in TNBC patients, prompting the investigation of therapeutic strategies targeting the inhibition of these enzymes 49.

In the treatment of TNBC, PARP inhibitors such as Olaparib (Lynparza) and Talazoparib (Talzenna) have been FDA-approved for patients with BRCA1/2 epigenetic alterations, offering a novel approach by targeting BRCA-related DNA repair mechanisms 49. These PARP inhibitors block the repair pathways of BRCA-induced DNA damage, leading to cancer cell death while simultaneously reducing treatment-related side effects when combined with chemotherapy 50. Additionally, DNA methyltransferase inhibitors (DNMTi), such as Azacitidine and Decitabine, have been approved by the FDA for treating hematological malignancies like acute myeloid leukemia (AML) and myelodysplastic syndrome (MDS), but their clinical efficacy in TNBC patients remains under investigation 51. However, the combination therapy of DNMT inhibitors and PARP inhibitors is currently under clinical investigation for TNBC patients with BRCA1/2 epigenetic alterations 52, offering a promising avenue for addressing the limited treatment options in TNBC 53, 54.

## 5. Histone Modifications in TNBC

**Figure 4:**
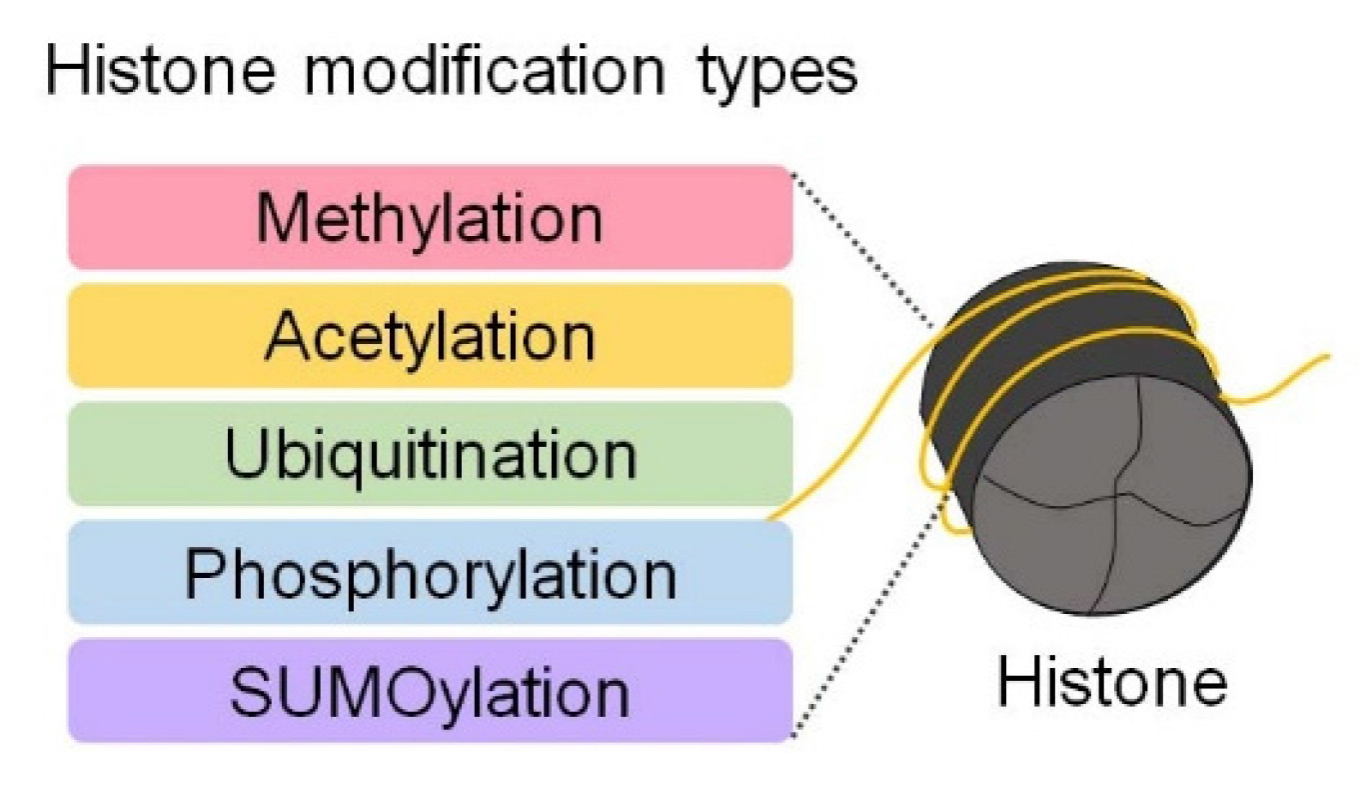
Post-translational modifications of histones. Histones undergo post-translational modifications, such as methylation, acetylation, ubiquitination, phosphorylation, and sumoylation. These modifications alter chromatin structure and accessibility, thereby regulating gene expression.

DNA is wrapped around histone proteins, forming nucleosomes, which are the basic units of chromatin 55. Nucleosomes are composed of a complex of eight histone proteins that package approximately 3 billion base pairs of DNA into a compact structure 55, 56. Modifications on histone tails induce subtle changes in chromatin architecture, serving as key mechanisms for regulating various gene expression processes 55, 56 (Figure 4). Histones undergo various chemical modifications, including methylation, acetylation, ubiquitination, phosphorylation, and sumoylation, depending on the specific amino acid residues involved 56. These modifications are tightly regulated by enzymes such as histone acetyltransferases (HATs), histone deacetylases (HDACs), and histone methyltransferases (HMTs) 56 (Figure 4). These modifications directly influence processes such as gene activation or repression, DNA repair, and replication 56. Acetylation loosens chromatin structure, facilitating transcription factor access to DNA and thereby promoting gene expression 56. In contrast, methylation compacts chromatin at specific gene regions, restricting transcription factor access and repressing gene expression 56.

Xi Y *et al.* conducted histone modification profiling on 13 TNBC cell lines, including MDA-MB-342, MDA-MB-436, MDA-MB-468, and HCC1937, focusing on eight types of histone modifications (H3K4me1, H3K4me3, H3K9me3, H3K9ac, H3K27me3, H3K27ac, H3K36me3, and H3K79me2) 57. Among these, a distinct pattern of H3K36me3 was observed 57. Notably, claudin-low TNBC cell lines exhibited the most active androgen receptor (AR) pathway gene expression, whereas basal-like cell lines showed low activity of AR pathway genes 57. These findings indicate that histone modification patterns vary among TNBC subtypes. Although the mechanisms of histone modifications in TNBC are not yet fully understood, therapeutic approaches targeting these features have shown promising results in preclinical studies 58-60.

The most widely studied epigenetic therapies targeting histone modifications are based on histone deacetylases 15. HDACs are enzymes that regulate gene expression by removing acetyl groups from histones, and their inhibition has been a focus of research through the development of histone deacetylase inhibitors (HDACis) 15. HDAC inhibition increases global acetylation levels on lysine residues of histone H3 and H4, particularly at the promoters of tumor suppressor genes such as CDKN1A (encoding p21), BAX, and BIM, leading to chromatin relaxation and enhanced transcriptional activation. This chromatin remodeling enables re-expression of genes that mediate cell cycle arrest (e.g., p21) and intrinsic apoptotic pathways (e.g., BAX, BIM), shifting the balance towards apoptosis in TNBC cells. Moreover, HDAC inhibition suppresses oncogenic signaling by downregulating anti-apoptotic genes such as BCL2 and survivin, and reducing the expression of EMT-related transcription factors such as SNAIL and TWIST, thereby limiting invasion and stemness properties. In TNBC cell line studies, HDACis such as vorinostat and sodium butyrate have been shown to suppress cell proliferation, induce apoptosis, and inhibit the transcription of mutant p53 in MDA-MB-231 and BT-549 cell lines 61. Specifically, HDAC inhibition in these models is accompanied by upregulation of p21 and p53-responsive genes, increased caspase-3 and caspase-9 activation, and cleavage of PARP, hallmarks of apoptotic cell death in response to chromatin de-repression 62, 63. Furthermore, in an MDA-MB-231 mouse model overexpressing PTEN, vorinostat demonstrated enhanced anti-proliferative effects when combined with the PARP inhibitor Olaparib 64.

Another HDAC inhibitor, panobinostat, has been shown to induce hyperacetylation of histones H3 and H4 in MDA-MB-157, MDA-MB-231, MDA-MB-468, and BT-549 cell lines, leading to decreased cell survival and proliferation 65, 66. hSETD1A, a histone methyltransferase involved in histone modification, has been associated with increased cancer aggressiveness and reduced survival rates in a retrospective study involving 159 TNBC patients 67. This suggests hSETD1A as a potential prognostic marker in TNBC progression.

## 6. Silencing of Non-Coding RNA in TNBC

RNA was traditionally regarded solely as a molecule involved in protein synthesis 68. However, the discovery of non-coding RNA has unveiled its critical role in the onset and progression of various diseases 68. Unlike protein-coding RNAs, ncRNAs are functional RNA molecules involved in critical physiological processes, including the regulation of gene expression, chromatin structure formation, and cellular signaling 16. ncRNAs are broadly classified into micro RNAs (miRNAs), which consist of fewer than 30 nucleotides, and long non-coding RNAs (lncRNAs), which consist of over 200 nucleotides 69.

miRNAs primarily regulate specific gene expression, potentially promoting or suppressing tumor growth 70. In contrast, lncRNAs operate in both the cytoplasm and the nucleus, functioning either in specific locations or shuttling between compartments 71. LncRNAs contribute to essential cancer-related processes, such as tumor proliferation, growth suppression, and angiogenesis, with over 50,000 identified 72.

The most recently identified epigenetic mechanism is ncRNA-mediated gene silencing 16. miRNAs regulate gene expression by degrading mRNA or inhibiting translation, whereas lncRNAs control gene expression at the transcriptional level through various mechanisms 16. Particularly in refractory cancers like TNBC, miRNAs play a crucial role in modulating chemotherapy resistance 16. This process involves diverse mechanisms, including DNA repair, autophagy, epithelial to mesenchymal transition (EMT), and cancer stem cell regulation 73.

In TNBC, proteins such as BRCA1 and FEN1 repair DNA damage, enabling cancer cells to evade the effects of chemotherapy 73. A reduction in miR-638 promotes excessive activation of BRCA1, increasing resistance to cisplatin (DDP) 74, while miR-140 suppresses FEN1 activity, enhancing the efficacy of Adriamycin (ADR) treatment 75. Furthermore, decreased expression of miR-489 induces ADR-triggered autophagy, allowing cancer cells to evade chemotherapeutic effects 76. Conversely, activation of miR-489 inhibits autophagy and enhances drug sensitivity 76. Additionally, miRNAs regulate EMT and cancer stem cell properties, both of which are associated with increased invasiveness and chemoresistance in cancer cells 73. For instance, miR-21-5p promotes EMT, leading to resistance against paclitaxel (PTX) 77, whereas miR-33a-5p suppresses EMT, enhancing the therapeutic efficacy of doxorubicin (DOX) 78. Regarding cancer stem cell characteristics, reduced levels of miR-29b-1-5p and miR-137 activate stem cell-related genes, such as SOX2 and NANOG, thereby promoting chemoresistance 79.

LncRNAs also play a crucial role in conferring chemoresistance in TNBC. LncRNAs H19 and LINP1 inhibit apoptosis-related proteins, increasing resistance to chemotherapeutic agents such as PTX and DOX, while HCP5 induces resistance to DDP by suppressing PTEN and activating the Akt pathway 80. Moreover, EV-packaged lncRNA HISLA stabilizes HIF-1α, promoting glycolysis and further enhancing chemoresistance 81. lncRNA-ROR promotes EMT, and NEAT1 enhances cancer stem cell properties, both contributing to increased resistance to chemotherapy, whereas decreased expression of TUG1 is associated with reduced resistance 81.

ncRNAs in TNBC serve as key regulators of chemoresistance, either promoting cancer cell survival or enhancing therapeutic efficacy through their unique structures and mechanisms. Therefore, ncRNAs hold significant potential as therapeutic targets for modulating cancer cell behavior and overcoming chemoresistance in TNBC.

## 7. Conclusion

The regulation of gene expression plays a pivotal role in the onset and progression of cancer, with epigenetic alterations providing critical insights into carcinogenesis and the development of novel therapeutic strategies. While epigenetic-targeted therapies present promising opportunities, substantial challenges must be overcome before they can be widely applied in clinical settings. TNBC, characterized by its aggressive and invasive progression and the absence of biomarkers for targeted therapy, currently has limited FDA-approved treatment options. Nevertheless, the identification of epigenetic mechanisms associated with TNBC has spurred research aimed at regulating these processes, and recently approved drugs hold promise for improving TNBC patient outcomes. Continuous monitoring of emerging research and clinical trials is essential. Recent advances include the development of more selective HDAC inhibitors and novel DNMT inhibitors, as well as approaches combining epigenetic drugs with immunotherapies or PARP inhibitors to enhance efficacy in TNBC. However, significant gaps remain in understanding the long-term effects, resistance mechanisms, and patient-specific epigenetic heterogeneity, which limit the translation of preclinical findings into clinical success. Furthermore, technological limitations in accurately mapping and quantifying dynamic epigenetic changes in patient samples present additional barriers to personalized therapy development. Addressing these challenges through integrative multi-omics approaches, improved delivery systems, and large-scale clinical validation will be crucial in advancing epigenetic therapies for TNBC. By deepening our understanding of these epigenetic changes and utilizing them to develop TNBC-specific therapies, innovative diagnostic and treatment options could be made available to significantly improve the prognosis and quality of life for TNBC patients.
